# Clinician and patient perceptions around implementing remote blood pressure monitoring for hypertensive disorders of pregnancy: A survey-based study

**DOI:** 10.1177/20552076251317567

**Published:** 2025-07-22

**Authors:** Theepika Rajkumar, Sharon Hu, Annemarie Hennessy, Angela Makris

**Affiliations:** 1 School of Medicine, 6489 Western Sydney University, Sydney, NSW, Australia; 2 Department of Medicine, 63608 Campbelltown Hospital, South Western Sydney Local Health, Liverpool, NSW, Australia; 3 Department of Renal Medicine, 1511 South Western Sydney Local Health District, Liverpool, NSW, Australia; 4 7800 University of New South Wales, NSW, Australia

**Keywords:** Antenatal, hypertensive disorders of pregnancy, pregnant women, remote blood pressure monitoring, questionnaire, survey

## Abstract

**Objective:**

Remote blood pressure monitoring (RBPM) is a promising method for surveilling hypertensive disorders of pregnancy (HDP). This refers to an organised framework in which clinicians can review and manage patient-obtained blood pressure recordings. Successful integration of RBPM into routine antenatal care necessitates understanding patient and clinician perspectives of its use. This study sought to evaluate the perceptions of high-risk pregnant women and clinicians regarding RBPM's use in HDP surveillance.

**Methods:**

A web-based questionnaire was distributed to pregnant women in South Western Sydney and clinicians across Australia and New Zealand. Quantitative Likert scales and qualitative open-ended questions were used to ascertain perceptions about RBPM for the surveillance of HDP.

**Results:**

Seventy-six women responded to the patient survey, with a response rate of 74.5%. A total of 65 clinicians responded to the healthcare professional survey. Almost all women (97.4%) supported RBPM's potential to aid healthcare decisions, with 96.1% willing to incorporate RBPM into antenatal care. Linear regression revealed implementation of RBPM was particularly supported by women who had less time off from paid employment during their pregnancy (*p* < 0.001). Conversely, women from non-English-speaking backgrounds and lower-income groups perceived RBPM as more challenging to use (*p* = 0.011 and *p* = 0.017, respectively). Most clinicians were concerned about the security of data transfer and storage interfaces. Poor accuracy or reliability of the technology emerged as a common barrier amongst both women and clinicians. Free-text responses revealed novel themes including accountability of care and medicolegal ramifications.

**Conclusion:**

Though socioeconomic status, ethnicity and language influenced perceptions towards RBPM; overall, Australian pregnant women and clinicians are broadly receptive to its use in antenatal care. Successful implementation of RPBM requires careful evaluation of design and workflow to accommodate for patient diversity, ease of use, compliance, privacy and clinical utility to optimise the user experience.

## Introduction

Preeclampsia affects 1 in every 20 women globally.^1^ Women with chronic hypertension, kidney disease, pre-existing diabetes mellitus, a previous hypertensive disorder of pregnancy, particularly preeclampsia, and autoimmune diseases^2,3^ are considered at high risk for developing preeclampsia. This high-risk population has an associated higher rate of adverse fetal outcomes (intrauterine growth restriction or preterm delivery) between 15% and 17%.^4^ The long-term maternal effects of preeclampsia are increasingly recognised as a greater risk of ongoing or future chronic hypertension, coronary artery disease, renal disease and stroke.^3,5–7^

Pregnant women at high risk for developing preeclampsia are recommended to undertake frequent blood pressure (BP) monitoring for early treatment, optimisation and detection of preeclampsia. This increased surveillance is undertaken with extra outpatient visits, compared to a lower-risk pregnancy, and usually includes review by a high-risk midwife, obstetrician, or hypertension specialist. The extra appointments, which are in addition to their routine antenatal schedule,^8^ expends significant resources at the level of both the patient and healthcare system. Despite this increased surveillance, preeclampsia can manifest between outpatient visits, leading to delayed instigation of appropriate treatment.

Following the COVID era, there has been widespread uptake of mobile health (mHealth) to address gaps in healthcare access.^9,10^ mHealth is defined by the World Health Organization as medical and public health practice supported by mobile devices, such as mobile phones, patient monitoring devices, personal digital assistants and other wireless devices.^11^ One such model of care that has been increasingly described is remote blood pressure monitoring (RBPM), particularly in the surveillance and management of high-risk pregnant women. Remote blood pressure monitoring refers to an organised framework that allows clinicians to review home-based blood pressure readings, institute management and provide participants with clear instructions when home blood pressure readings are out of prespecified targets. There are variable definitions of RBPM within the literature, including manually written BP logs^12–15^ with patients notifying clinical staff via phone calls of BP out of prespecified targets,^16,17^ BP readings sent via text message^18–20^ and manual or automatic upload of BP readings to web-based dashboards via mobile applications.^12–15,21–28^ Review of digitally transmitted BP readings are predominantly asynchronous by nursing staff, midwives or doctors,^12–15^ while some mobile application-based frameworks of RBPM may provide immediate automated responses based on BP readings.^21,24^

While existing cohort studies have indicated promise for the use of RBPM in the antepartum and postpartum periods,^29–32^ findings from large randomised controlled trials are mixed. The BUMP trials^12,15^ showed that RBPM did not lead to earlier detection of hypertension or improved blood pressure control in women at higher risk of developing preeclampsia, while ‘POP-HT’^33^ showed that RBPM utilised postpartum in women with hypertensive disorders of pregnancy led to lower blood pressures in the long-term.

However, prior to the widescale application of RBPM, it's important to ascertain an understanding of patient and clinician perceptions of the technology. Existing data (United Kingdom, Belgium, the Netherlands and the USA) show that high-risk women found RBPM easy to use, preferring it over conventional clinic BP measurements, and would recommend it to other high-risk pregnant women.^22,34^ However, limitations that may increase patient hesitation to adopt RBPM include perceived technological barriers, heightened vigilance and safety and privacy concerns.^35,36^ From the clinician perspective, midwives and obstetricians alike understand the utility of RBPM,^36,37^ yet have concerns regarding accuracy of readings and inaction by patients.^38^

In this study, we aim to explore the perspectives of Australian women with pregnancies at high risk of preeclampsia, along with midwives and physicians involved in their care. We aim to attain insights into facilitators or barriers to implementing RBPM in obstetric care, to inform the implementation of the REMOTE CONTROL™ trial, the first Australian randomised controlled trial evaluating RBPM.^39^ These results may differ from previously published results due to Australia's unique social and geographical considerations. Women must travel significant distances for review given the distribution of the population and health services in Australia as well as the unique universal healthcare system in place. We hypothesise that patients and clinicians will welcome the implementation of RBPM and have diverse perceptions, influenced by demographics, context and healthcare system factors.

## Methods

### Recruitment

Two separate cross-sectional web-based surveys were conducted to (1) pregnant women at high risk for developing preeclampsia and (2) healthcare professionals involved in the care of these pregnant women.

Pregnant women were recruited from the high-risk outpatient antenatal clinics of Campbelltown Hospital, Liverpool Hospital and Bankstown Hospital, within South Western Sydney Local Health District (SWSLHD) (Sydney, Australia), between June 2022 and October 2023. At these clinics, pregnant women, who have an HDP or are at high risk of developing an HDP according to the United Kingdom (UK) National Institute for Health and Care Excellence (NICE) guidelines, are reviewed from 12 weeks’ gestation onwards.^2^ Convenience sampling was undertaken, based on the availability of the research team to attend the scheduled clinics across the three sites, but all eligible women were approached within each sampled clinic. Women were approached to participate by a medical student (SH). Consenting women were either provided with a QR code which allowed the survey to be completed on their smartphone, completed the survey on a research tablet device or were offered a paper survey if they preferred. A member of the research team was present to answer any queries but was not present when the participant completed the survey. Healthcare professionals across Australia and New Zealand who were involved in the care of high-risk pregnant women (specifically midwives, obstetricians and obstetric medicine physicians) were also invited to participate. Healthcare professionals at the SWSLHD hospitals were approached through email correspondence from the obstetrics and gynaecology department, with a web link or QR code to the survey. Other clinicians were reached through email correspondence from a professional organisation, the Society for Obstetric Medicine of Australia and New Zealand (SOMANZ). No reminder or follow-up emails were sent to clinicians contacted via email. Midwives at the SWSLHD hospitals were provided with either a QR code to the questionnaire survey or a printed copy to be filled out manually. These were distributed by a research midwife at midwifery departmental meetings. Healthcare staff were recruited between June 2022 and August 2023.

All respondents completed surveys prior to recruitment, involvement or receiving information about the REMOTE CONTROL trial.^39^ As non-probability sampling was undertaken for this exploratory work, a sample size calculation was not undertaken. However, recruitment was concluded when open-ended answers reached thematic saturation. Ethics approval was granted by the SWSLHD human research ethics committee (HREC) (approval number 2021/ETH00528), and all members of the research team had a Good Clinical Practice (GCP) certificate.

The inclusion criteria for this study were as follows:

1. Pregnant women who are classified as being at high risk of developing an HDP, as per the United Kingdom's National Institute for Health and Care Excellence (NICE) guidelines^40^ or who already have a diagnosis of a HDP.
2. Clinicians who are involved in the care of pregnant women, including midwives and doctors.

Pregnant women with low-risk pregnancies, less than 16 years of age or who were unable to consent were excluded. Participants eligible for inclusion in this study represent the target user population for RBPM.

### Survey development

The surveys were developed by the investigators, obstetric medicine physicians within SWSLHD. The survey was developed without the involvement of any patient partners. Surveys collected self-identified demographic data before presenting respondents with statements on themes related to the utilisation of RBPM. Themes and statements were generated based on a literature review of previous publications.^38,41–45^ To respond to these statements, a five-point Likert scale (1 ‘strongly disagree’ to 5 ‘strongly agree’) was utilised. For the patient survey, there were nine separate questions, and the clinician survey was composed of 10 separate questions. Questions assessed women and healthcare professionals’ perceptions of (1) ease of mobile application, technology and device usage, (2) privacy and security and (3) clinical value of RBPM. Participants were then asked to optionally select from a list of possible barriers to using RBPM, based on previous work.^38,41–45^ A final open-ended question encouraged written responses on any perceived barriers that were not covered in the survey prior. The full-text version of both surveys is included in the Supplemental material. Surveys were administered in English only, through REDCap™.^46^ Participants without sufficient English skills to complete the survey were excluded and English was compared to all other languages as a group. The survey was not pilot-tested prior to use in this study.

### Data collection and statistical analysis

Data from manually completed surveys were entered into REDCap™ by a member of the research team who was not involved in the final statistical analysis. Data analysis was conducted using IBM SPSS Statistics v27. Likert scale responses were treated as a continuous variable for analysis. A combination of descriptive statistics (mean ± standard deviation (SD) for normally distributed data; median ± IQR (interquartile range) for non-normally distributed data) and multiple linear regression analysis was employed. Linear regression was utilised in post hoc analysis of associations between women's and clinicians’ characteristics and survey responses. Free-text responses were coded using a reflexive thematic analysis approach in which some common categories were identified across items.

## Results

### Pregnant women

A total of 102 high-risk pregnant women were approached to participate in the study. Seventy-six women across all sites responded to the patient survey, with a response rate of 74.5%. Table 1 outlines the characteristics of the survey respondents. Women were more likely to be multiparous, and the majority (63.2%) were in paid employment, of which most were offered time off to attend antenatal appointments. The cohort was ethnically diverse, and more than half of the participants (51.3%) did not speak English as their first language.

**Table 1.** Characteristics of pregnant women.

Characteristic	
Age, years ; median (±IQR)	34 (±6)
Weight, kilograms; median (±IQR)	89.3 (±30)
Height, cm; mean (±SD)	162 (±0.11)
Body mass index, kg/m^2^; mean (±SD)	33.6 (9.3)
Gravidity; mean (± SD)	3 (±2)
Parity; mean (± SD)	1 (±1)
Gestation, weeks; median (± IQR)	24 (±15)
Ethnicity; n (%)	
Asian	29 (38.2%)
Caucasian	21 (27.7)
African or Middle Eastern	19 (25)
Native, Central or South American Aboriginal/Torres Strait Islander	4 (5.3%)
Pacific Islander	2 (2.6)
	1 (1.3)
First language; n (%)	
English	37 (48.7%)
Arabic	4 (5.3%)
Vietnamese	3 (3.9%)
Other	32 (42.1%)
Married; n (%)	63 (82.9)
Time off for appointments [yes] ; n (%)	35 (72.9%)
Annual gross income [$AUD]; n (%)	
<$20,000	24 (31.6%)
$20,000–$50,000	14 (18.4%)
>$50,000	38 (50.0%)
Hospital site; n (%)	
Campbelltown	29 (38.2%)
Liverpool	28 (36.8%)
Bankstown	19 (25.0%)
Employment status; n (%)	
Studying	1 (1.3%)
Paid employment	48 (63.2%)
Independent entrepreneur	1 (1.3%)
Housewife	17 (22.4%)
Unemployed	9 (11.8%)
Highest level of education (n; %)	
Secondary education or lower	16 (21.1%)
Tertiary education	60 (78.9%)
Method of conception for current pregnancy (n; %)	
Natural	71 (93.4%)
In-vitro fertilisation	5 (6.6%)

AUD: Australian dollar; n: number; SD: standard deviation; IQR: interquartile range.

**Figure 1.**
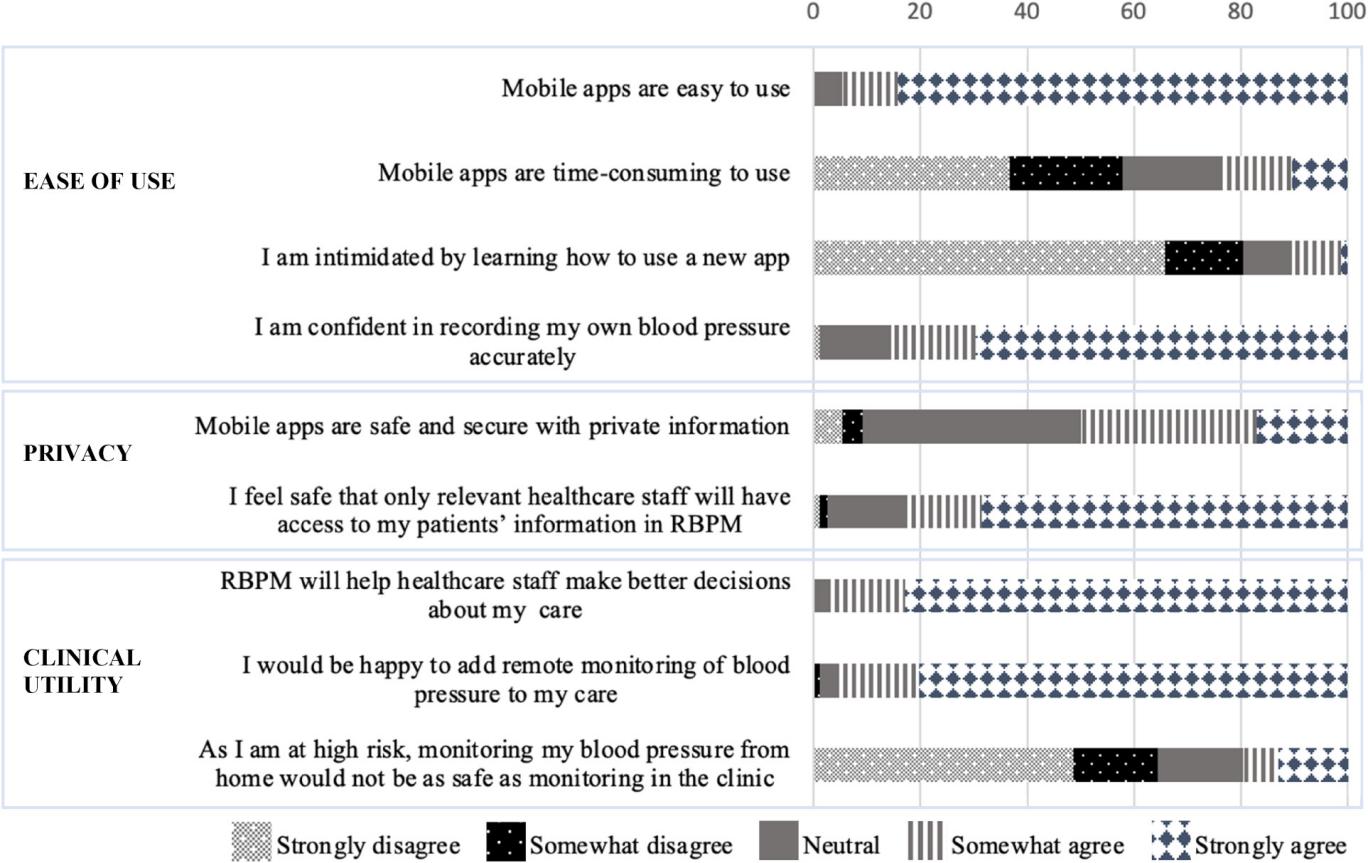
RBPM survey responses – pregnant women.

Pregnant women's responses regarding mobile technologies and RBPM are outlined in Figure 1.

Most women (94.7%; 72/76) perceived mobile applications to be easy to use. A quarter (23.7%; 18/76) of women felt they may be time-consuming, and 10.5% (8/76) of women were intimidated by learning how to use a new mobile application. Only one woman (1.3%) did not feel confident measuring her own blood pressure. Most women (82.9%; 63/76) felt safe that only healthcare staff would have access to health information through a RBPM framework, while only 9.2% (7/76) of women felt mobile applications were not safe and secure with private information. Regarding clinical utility, 97.4% (74/76) of women thought RBPM would help their healthcare team make better decisions about their care, and 96.1% (73/76) were happy to add RBPM to their antenatal care. However, 19.8% (15/76) of women felt that given they were at high risk of developing preeclampsia, only monitoring their blood pressure at home was not as safe as conventional clinic monitoring.

**Figure 2.**
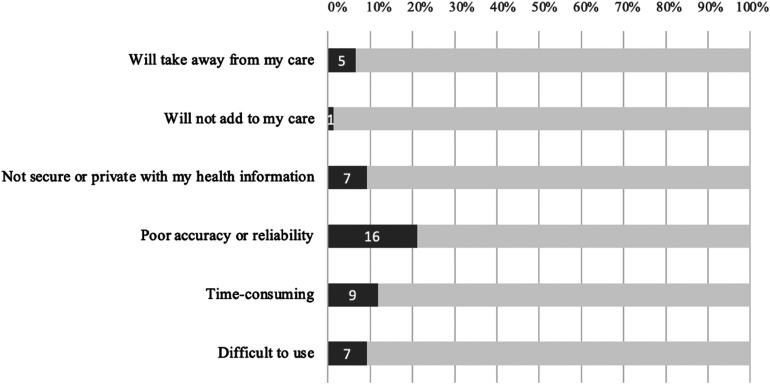
Pregnant women's perceived barriers to RBPM implementation.

Most respondents (78.9%; 60/76) did not select or suggest any perceived barriers to usage. Figure 2 outlines the potential barriers selected by pregnant women.

Only nine women (11.8%) provided open-ended responses to perceived barriers. These answers revealed women were concerned about *forgetting to record their BP*, particularly whether it would fit in with their lifestyle. Others thought that the technology and devices inherent to RBPM may require a certain skill set and be *challenging to use**.*** An *increase in health-related anxiety* was also raised as a potential barrier to uptake, such as patients struggling with what to do if they were to record an abnormal blood pressure measurement at home. Another important consideration that was identified was that a lack of face-to-face interaction with healthcare staff may lead to *uncertainty in the quality of care being received*.

Other open-ended barriers that were suggested included failure of technology, cost, language barriers restricting use and security of health information. The free-text responses to support each theme are outlined in Table 2.

**Table 2.** Women's free-text responses regarding perceived barriers to remote blood pressure monitoring.

Perceived barriers	Free-text responses
Poor patient compliance	‘Could be difficult to remember to do it consistently’. – W1
	‘If the measurement taking conflicts with [my] work schedule and I forget’. – W6
Challenging to use	‘Self-doubt in whether [I am] using it properly’. – W2
	‘If I am not equipped with skills’. – W9
Increase in health-related anxiety	‘Could make me anxious to be hyper-aware of high blood pressure with nobody at home to necessarily help with that’. – W4
Connectivity and access issues	‘If there were difficulties in connectivity to Wi-Fi/Bluetooth, which would impact utility’. – W7
Uncertainty in the quality of care being received	‘If someone who is not qualified evaluates my data’. – W5
Security	‘Security of my information’. – W10
Language constraints	‘Language barrier in understanding the usage’ – W8

Post hoc, all demographic characteristics were tested for statistically significant associations. Those found to be statistically significant are summarised in Table 3. Post hoc analysis revealed that in the *ease-of-use domain*, a higher BMI (*p* = 0.012) was associated with the perception that mobile apps are easy to use. A higher annual gross income and identifying English as their first language were predictive of not being intimidated by learning a new app (*p* = 0.011 and *p* = 0.017, respectively). Those identifying English as a first language were also less likely to perceive mobile apps as time-consuming (*p* = 0.028).

**Table 3.** Association between characteristics of pregnant women respondents and survey responses.

Domain	Dependent variable	Explanatory variables	Coefficient (standard error)	95% CI	Characteristic with higher Likert scores (1 = strongly disagree; 5 = strongly agree)	*p*-value
**Ease of use**	Mobile apps are easy to use	BMI	0.016 (0.006)	0.004–0.029	Higher BMI	0.012
	Mobile apps are time-consuming	First language	0.684 (0.304)	0.078–1.289	Non-English-speaking background	0.028
	I am intimidated by learning how to use a new app	Annual income	−0.336 (0.129)	−0.593 to −0.080	Lower annual income	0.011
		First language	0.559 (0.228)	0.105–1.013	Non-English-speaking background	0.017
**Privacy and security**	Mobile apps are safe and secure with private information	Annual income	−0.263 (0.126)	−0.515 to −0.011	Higher annual income	0.041
		Parity	−0.215 (0.097)	−0.409 to −0.012	Lower parity	0.030
**Clinical utility**	RBPM will help healthcare staff make better decisions about my care	Time off from paid employment	−0.012 (0.002)	−0.016 to −0.007	Less time off from paid employment	<0.001
	I would be happy to add RBPM to my care	Time off from paid employment	−0.012 (0.002)	−0.015 to −0.009	Less time off from paid employment	<0.001

With regard to *privacy and security*, lower income (*p* = 0.041) and higher parity (*p* = 0.030), when adjusted for age, were associated with more concern for the safety and security of private information utilised by mobile applications.

Pregnant women with less time off from paid employment were more likely to see the *clinical utility of RBPM*. Specifically, they perceived that RBPM would help healthcare staff make better decisions about their care (*p* < 0.001) and were more likely to agree to add RBPM to their antenatal care (*p* < 0.001).

Regarding perceived barriers, women who were unemployed were more likely to perceive RBPM as potentially difficult to use (*p* = 0.02). Poor accuracy and reliability were more likely to be perceived as a barrier in women who had more time off from work (*p* = 0.03).

### Clinicians

A total of 65 clinicians responded to the healthcare professional survey, with their demographic characteristics outlined in Table 4. As surveys to clinicians were also distributed through email correspondence with an unknown number of potential respondents, a response rate for clinicians is unable to be reported. Most respondents were female (87.9%) and midwives (60.6%). On average, participants had extensive experience caring for pregnant women, with a mean of 14.21 years. The cohort was ethnically diverse, with 36.4% not identifying English as their first language.

**Table 4.** Demographic characteristics of clinicians.

Characteristic	
Age, years; median (±IQR)	44 (±18)
Experience in the care of pregnant women, years; mean (± SD)	14.4 (±10)
Female gender; n (%)	57 (87.7)
Ethnicity, n (%)	
Aboriginal/Torres Strait Islander	2 (3.1)
Pacific Islander	2 (3.1)
Caucasian	32 (49.2)
African or Middle Eastern	7 (10.8)
Asian	19 (29.3)
Native, Central or South American	2 (3.1)
First language, n (%)	
English	41 (63.1)
Other	24 (36.9)
Role in the care of pregnant women, n (%)	
Obstetrician	8 (12.3)
Physician	13 (20.0)
Midwife	40 (61.5)
Undisclosed	4 (6.2)
Hospital site, n (%)	
Campbelltown	18 (27.7)
Liverpool	26 (40.0)
Bankstown	13 (20.0)
Outside South Western Sydney LHD	8 (12.3)
Level of medical training (n = 21) (%)	
Registrar	4 (19.0)
Consultant	16 (76.2)
Undisclosed	1 (4.8)

**Figure 3.**
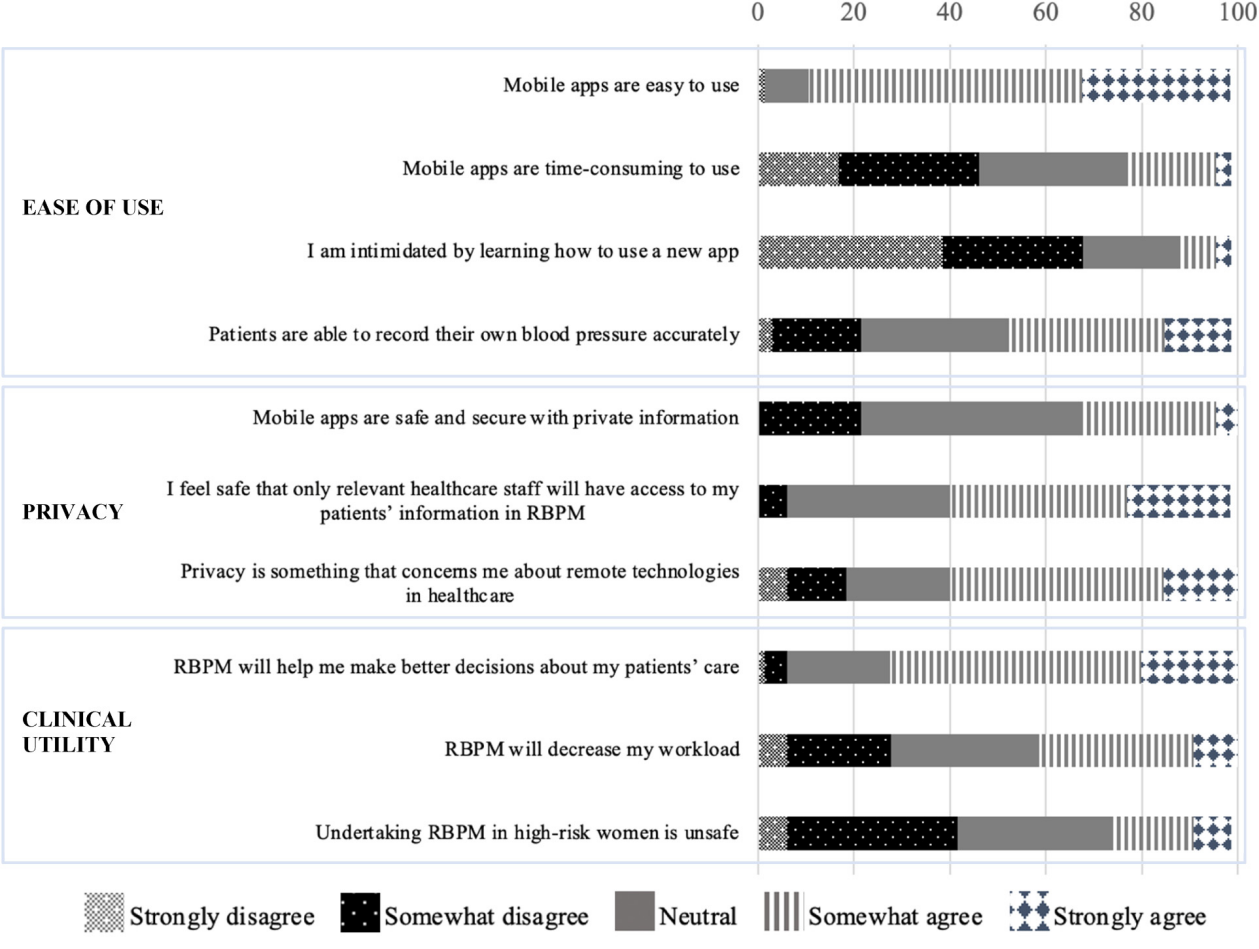
RBPM survey responses – clinicians.

Clinician responses to the survey are detailed in Figure 3.

Like surveyed women, most clinicians and midwives (87.7%; 57/65) perceived mobile applications to be easy to use. Only 21.6% (14/65) responded that mobile apps were time-consuming to use, and 9.2% (6/65) said they were intimidated by the prospect of learning to use a new mobile application. Amongst healthcare staff, 21.6% (14/65) were not confident in their patients’ capacity to record their own blood pressure accurately. Regarding privacy and security, 60% (39/65) of clinicians, had concerns about privacy in remote healthcare technologies. Yet, only 6.2% (4/65) were concerned about inappropriate access to patients’ information through RBPM, while 21.5% (14/65) felt mobile apps were not safe and secure with private information. In the domain of clinical utility, most respondents (72.3%; 43/65) saw the potential for RBPM to help make better decisions about patient care, with 41.5% (27/65) of healthcare staff anticipating a decrease in workload. Sixteen respondents (24.6%) were concerned that RBPM would be unsafe to institute in the care of high-risk women.

**Figure 4.**
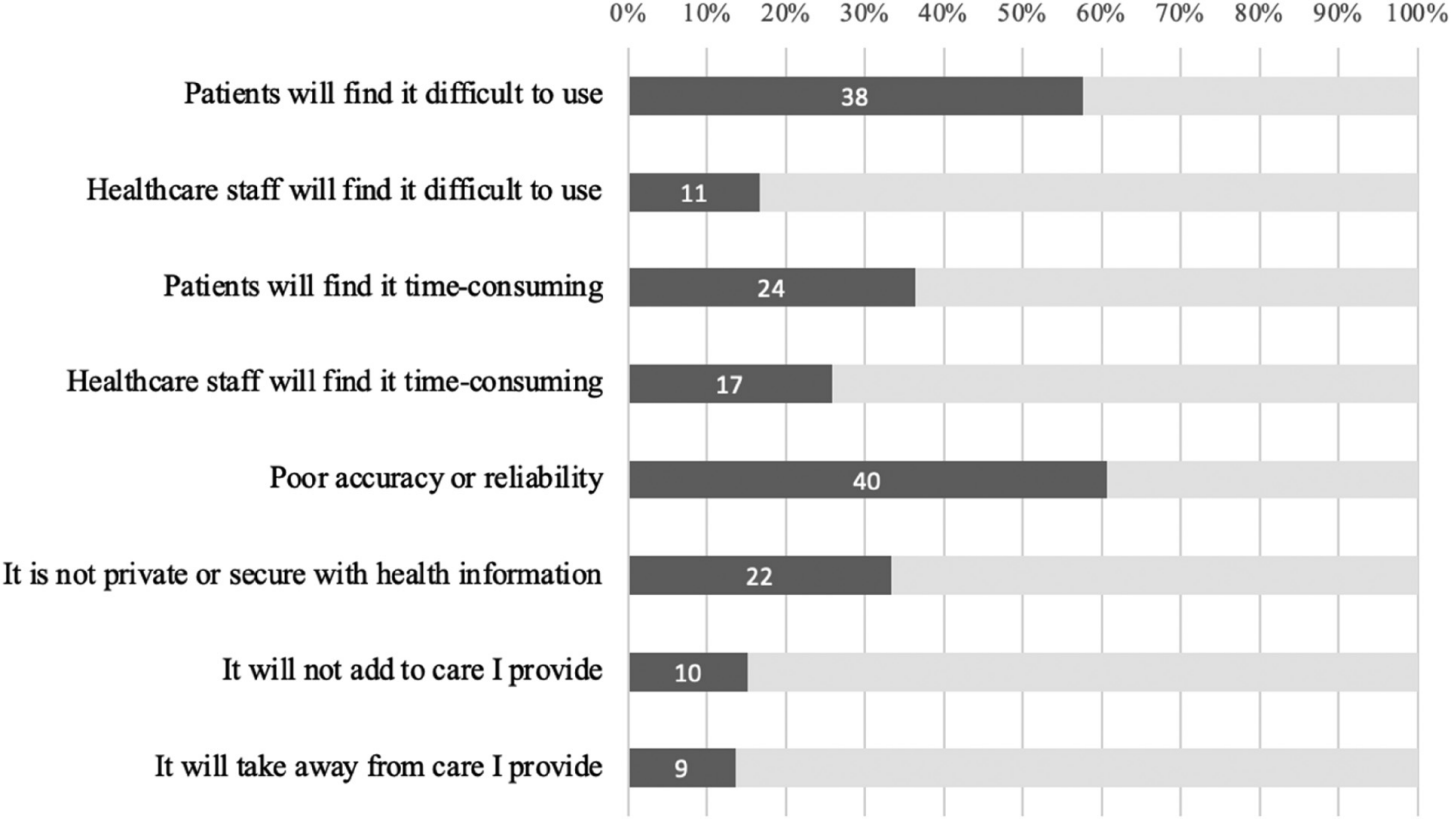
Clinicians’ perceived barriers to RBPM implementation.

All but five respondents (92.3%) selected a potential barrier to the institution of RBPM. Figure 4 summarises the barriers selected.

Twenty-seven clinicians (41.5%) had open-ended responses to possible barriers to RBPM implementation, outlined in Table 5. *Poor patient compliance* with RBPM was the most common concern. One midwife was concerned that patients would ‘falsify results’, while others recognised the importance of adequate education to overcome poor compliance, for example:

Compliance is always an issue but with adequate education can be achieved.

**Table 5.** Clinicians’ free-text responses regarding perceived barriers to remote blood pressure monitoring.

Perceived barriers	Free-text responses
Poor patient compliance	‘Some patients cannot be trust[ed] especially if they don’t want admission, we have seen that in [gestational diabetes mellitus]. Some patients won’t monitor at all. Some will do the BP and not record it with fear of admission’. – C8
	‘Compliance is always an issue…’ – C2
	‘General non-compliance’ – C4
	‘…patients not wanting to take responsibility of own care’. – C11
Inaccurate technique and measurements	‘Patients need education on taking good blood pressure’.
	‘Incorrect technique used by patients’ – C3
	‘Patients may use inadequate technique’ – C6
	‘Reliability, accuracy of results’ – C9
Need for calibrated, validated devices	‘Sphygmomanometers used by patients may not be regularly calibrated’. – C6
	‘Do they calibrate the monitor? Many machines are not accurate. Manual BP is more accurate in pregnant women’. – C10
	‘I would be checking it myself and digital BP machines are already more inaccurate than manual’. – C18
	‘BP monitor should be validated for use in pregnancy’. – C27
Patient's education level and language barriers	‘Clinical understanding for patients, whether they are educated enough to understand the BP readings well, or if know how to attend to BP check properly’. – C22
	‘Patient education level, [non-English-speaking background]’ – C11
	‘Lack of education might hinder patient from using app’. – C12
	‘Lack of health literacy among women. Increased risk of safety if inadequately attended/recorded’. – C17
	‘Issues with patients who are non-English-speaking. Increase health inequity by language and socioeconomic status…’ – C20
Accountability of care and medicolegal issues	‘As In BUMP2 trial self-monitoring of BP in [patients] with [chronic hypertension] or gestational [hypertension] did not lead to improved clinic-based BP control - the monitoring of BP is not the issue, but what is done with the information’. – C27
	‘Who would be responsible for checking and providing [follow-up] care - designated clinician would be preferred’. – C14
	‘If the patient has severe hypertension recorded at home there needs to be clear guidelines and instructions to avoid mismanagement and patient harm. Who will be monitoring remote BPs after hours?’ – C24
	‘Medicolegal issues about monitoring BP during out of working hours/over long holidays/when clinicians is away. This actually appears to increase the clinician's workload’. – C25
Connectivity and access issues	‘Not everybody has access to technology and data. We found with the young mums that they would love the technology care however they lack the tools usually only have phones with limited data so the uptake was not there’. – C5
	‘Reliable internet at home not possible for very low SES’ – C20
	‘Internet access’ – C19
	‘Poor technology’ – C23
Patients’ health-related anxiety	‘…Anxiety provoking for women with history’. – C7
	‘Over-recording and increasing patient anxiety’. – C21
Limited clinical utility in high-risk pregnancies	‘I think it would be good for well-controlled BP and normal bloods if it is implemented. I would worry about high risk’. – C13
	‘BP monitoring alone is not adequate when monitoring high-risk women. They also need growth scans, blood tests, etc. which will need visit[s] to clinics anyway’. – C26

The importance of patient education was again emphasised to overcome *inaccurate technique and measurements*, which was the second most cited barrier. For example:

Patients need education on taking good blood pressure.

In addition, multiple responses mentioned hesitation with using automatic blood pressure machines, doubting their accuracy, and the *need for using validated, calibrated devices* to improve reliability of readings.

Multiple issues with *accountability of care* were also identified. Some healthcare staff had hesitations about whether blood pressure readings attained through RBPM would be acted upon and therefore improve overall blood pressure control or whether there would be decision paralysis from clinicians. Additionally, there were concerns about whether RBPM could lead to *medicolegal issues* if abnormal readings prompted management to be provided outside of work hours and furthermore whether this would increase the antenatal team's workload.

Medicolegal concerns also extended to the use of technology in the healthcare setting, especially in terms of communicating with patients, for example:

Ensuring we are not replying on app.

Like the pregnant women respondents, many healthcare staff recognised that a *patient's education level and language barriers* may limit the utilisation of technology inherent to RBPM. Other technological concerns included *connectivity and access issues*, particularly for low socioeconomic populations. There were concerns that RBPM could also potentially increase *patient's health-related anxiety*, particularly for women who had previously had HDP and poor outcomes. Some midwives and clinicians felt that RBPM would have *limited clinical utility in high-risk pregnancies* and was more suitable for low-risk women. Specifically, in the context of high-risk women, they believed that RBPM was unlikely to be safe, nor would it reduce healthcare utilisation.

Post hoc, all demographic characteristics were tested for statistically significant associations. Those found to be statistically significant are summarised in Table 6. In the *ease-of-use* domain, older age of healthcare staff correlated with more confidence in their patients’ ability to record their own blood pressure accurately (*p* = 0.007). In terms of *privacy and security*, the belief that mobile applications are safe and secure with private information was also correlated with older age (*p* = 0.029). Amongst doctors, consultants had greater concern for privacy in remote technologies used for healthcare, compared to trainee doctors (*p* = 0.038).

**Table 6.** Association between characteristics of healthcare staff respondents and survey responses.

Domain	Dependent variable	Explanatory variable	Coefficient (standard error)	95% CI	Characteristic with higher Likert scores (1 = strongly disagree; 5 = strongly agree)	*p*-value
**Ease of use**	Patients are able to record their own blood pressure accurately	Age	0.032 (0.011)	0.009–0.055	Older age	0.007
**Privacy and security**	Mobile apps are safe and secure with private information	Age	0.020 (0.009)	0.002–0.038	Older age	0.029
	Privacy is something that concerns me about remote technologies in healthcare	Level of training	1.250 (0.559)	0.076–2.424	Trainee doctors	0.038
**Clinical utility**	RBPM will help me make better decisions about my patients’ care	First language	0.648 (0.214)	0.219–1.077	Non-English-speaking background	0.004
	RBPM will decrease my workload	First language	0.630 (0.291)	0.048–1.212	Non-English-speaking background	0.035
	Undertaking RBPM in high-risk women is unsafe	Gender	−0.856 (0.376)	−1.608 to −0.105	Male clinicians	0.026

Regarding *clinical utility*, male healthcare staff responded they did not perceive RBPM as unsafe in high-risk pregnancy (*p* = 0.026), compared to their female counterparts. Healthcare staff of non-English-speaking background felt RBPM could improve clinical decision-making (*p* = 0.004) and decrease their workload (*p* = 0.035), compared to their colleagues from an English-speaking background.

For clinicians, no demographic variables were significantly associated with perceived barriers to use.

## Discussion

### Principal results and comparison with prior work

This study is the first to assess pregnant women and clinicians’ perceptions before implementing an RBPM strategy for monitoring HDP, with both groups showing broad receptiveness to the RBPM approach. These findings will inform the implementation of the first randomised controlled trial in the Australian healthcare system to evaluate RBPM for high-risk pregnant women with preeclampsia risk.

Both pregnant women and clinicians expected RBPM to be user-friendly, but healthcare staff expressed less confidence in patients’ ability to accurately record their blood pressure. Various factors contribute to this concern, including non-adherence, incorrect technique, fabricated readings, use of uncalibrated devices and potential language barriers or education levels that may hinder RBPM use. Qualitative feedback underscores the need for comprehensive and standardised patient education to address these challenges. There are also valid concerns regarding the accuracy of devices, as only a limited number of monitors on the market are validated for use during pregnancy and for detecting preeclampsia.^47^

Regarding privacy, while mobile apps were overall deemed secure, almost two in three healthcare staff were concerned about remote technologies, such as the transmission protocols (Wi-Fi, Bluetooth or broadband) and remote platforms that store health information (the Cloud or web portals) While existing studies have reported on privacy concerns by patients,^45^ no studies have evaluated the privacy concerns of healthcare staff using RBPM. This is an important consideration as mHealth gains traction and is increasingly utilised by clinicians. Remote blood pressure monitoring incorporates connected devices and mHealth apps, which contain medical information with integration into backend systems that contain additional critical data, all potential targets for data breaches. Adoption of digital technologies requires implementation of appropriate safeguards for privacy of all data storage and data transfer interfaces.^48^

While most women and clinicians felt RBPM would improve clinical decision-making, almost a quarter of participants felt RBPM would not be as safe as conventional clinic monitoring for high-risk women. This finding implies that RBPM should be considered an adjunct to regular clinic visits, rather than a replacement. Hinton et al. explored the perceptions of English National Health Service staff towards home-based blood pressure monitoring in pregnancy and found they similarly would treat home readings as contributing extra detail to care decisions, rather than core information.^44^

Clinicians identified significantly more barriers than pregnant women, to the introduction of RBPM into clinical practice, including non-adherence, patients’ language and education limiting use, an increase in patients’ health-related anxiety, technological access and connectivity issues, inaccurate technique and uncalibrated devices, clinician indecision with varied blood pressure readings and accountability of care. These are consistent with previous published work,^42,44,45^ yet the medicolegal ramifications of RBPM were a novel issue that was raised in this study. Following the COVID-19 pandemic, there has been an expansion of telehealth; however, there have been challenges with the integration of these services into the health system, including service setup within existing pathways, the supply, management and return of monitors and managing follow-up.^49^ Furthermore, as identified by survey respondents, RBPM care may need to be provided after-hours or on weekends and public holidays, and this carries significant medicolegal implications. Stringent workflow and responsibilities need to be established prior to implementation, to balance patient safety with professional liability.

A unique finding of this study is the characteristics of both patients and clinicians which may act as facilitators or barriers to uptake of RBPM in clinical practice. In this study, pregnant women were diverse in level of education, socioeconomic status, ethnicity and first language. Of the surveyed pregnant women, those who identified from a non-English-speaking background and those with lower annual income were more likely to perceive aspects of RBPM as difficult to use. These findings are unsurprising, as there is a strong association between low health literacy and social determinants of health such as lower levels of education and socioeconomic status and being from a culturally and linguistically diverse background.^50–52^ People with low health literacy are more likely to have worse health outcomes overall^53^ and adverse health behaviours, including a lower ability to self-manage care.^54^ An essential aspect of RBPM is a level of patient autonomy and responsibility,^44^ and thus implementing novel digital health technologies requires consideration of this diversity, particularly of language, and cultural and socioeconomic barriers to access. This is integral to ensuring that RBPM in antenatal care lessens, rather than increases the ‘digital divide’. The impact of sociocultural factors also extends to healthcare staff, with clinicians from a non-English-speaking background expecting that RBPM could improve clinical decisions and decrease their workload. This may represent language barriers that non-English-speaking clinicians and midwives face in therapeutic interactions, which they envisage RBPM may overcome. Older age is a known barrier to the adoption of mHealth tools by patients^55^ and clinicians,^56^ and the findings of this study are consistent with this. Surprisingly, older clinicians were more likely to score RBPM higher in the ease-of-use and privacy domains, which is divergent from previous findings.^56^ Gender also influenced perceptions of RBPM, with male clinicians more optimistic about the clinical utility of RBPM than their female colleagues. This contrasts with a previous systematic review which reported gender had an inconsistent influence on mHealth adoption and usage by clinicians.^56^ However, men were underrepresented in this survey and this may be a confounder.

One benefit of home-based blood pressure monitoring reported in the literature is a reduction in outpatient appointments.^14,57^ Unsurprisingly in this study, women who had less time off from paid employment to attend appointments, were more positive about the institution of RBPM.

### Strengths and limitations

A significant strength of this study is the analysis of demographic and clinical characteristics of both women and healthcare staff, which revealed factors that may act as facilitators or barriers to the uptake of RBPM into clinical practice, an important aspect of translational research. The surveyed women were diverse in age, parity, language, ethnicity, education level and socioeconomic status, increasing the generalisability of the findings. Furthermore, the surveyed clinicians and midwives were also ethnically diverse, from both South Western Sydney, and across Australia and New Zealand. The use of a mixed methods design, including Likert scale responses, as well as open-ended responses is also a strength of this study. The use of quantitative and qualitative data provides a more rounded assessment of the perceptions towards RBPM, adding depth and breadth to this study. Additionally, this study included all stakeholders involved in the use of RBPM, including healthcare providers and patients, prior to implementation, so that expectations can be compared with user experience after institution.

All surveyed sites were metropolitan hospitals and therefore limit applicability for rural and regional sites, where RBPM may be of greatest benefit. The survey was only administered in English which introduces selection bias. A further source of selection bias arises from the preferential administration of the survey in an electronic format, as highlighted previously. Another potential limitation is that while the survey questions were developed by the research team, they were not validated prior to use. This may reduce reliability as an instrument; however, questions were paired with Likert ordinal scale responses and appropriately analysed using non-parametric testing, reducing the likelihood of inaccurate results.

The findings of this study are crucial to the success of implementing a RBPM strategy for surveillance of HDP in high-risk pregnant women. Utilising the findings of this study to develop a cost-effective RBPM framework and evaluate its safety and efficacy in the context of a clinical trial would be the next steps forward.

### Conclusions

The institution of RBPM for the surveillance and management of hypertensive disorders of pregnancy requires consideration of the context, stakeholders and healthcare system.

Though numerous factors such as socioeconomic status, ethnicity, language, age and gender influenced perceptions of both women and healthcare staff; overall, Australian pregnant women and clinicians are broadly receptive to the potential introduction of RBPM, predicting it will be a useful clinical tool that will be easy to use. Some women and healthcare staff felt RBPM should only be an adjunct to clinic monitoring. Additionally, healthcare staff were concerned about the privacy of health information when transferred and stored remotely from mobile applications.

Successful implementation of RPBM requires careful evaluation of design and workflow to accommodate for patient diversity, ease of use, compliance, privacy and clinical utility to optimise the user experience.

## Supplemental Material

sj-docx-1-dhj-10.1177_20552076251317567 - Supplemental material for Clinician and patient perceptions around implementing remote blood pressure monitoring for hypertensive disorders of pregnancy: A survey-based study Supplemental material, sj-docx-1-dhj-10.1177_20552076251317567 for Clinician and patient perceptions around implementing remote blood pressure monitoring for hypertensive disorders of pregnancy: A survey-based study by Theepika Rajkumar, Sharon Hu, Annemarie Hennessy and Angela Makris in DIGITAL HEALTH

sj-docx-2-dhj-10.1177_20552076251317567 - Supplemental material for Clinician and patient perceptions around implementing remote blood pressure monitoring for hypertensive disorders of pregnancy: A survey-based study Supplemental material, sj-docx-2-dhj-10.1177_20552076251317567 for Clinician and patient perceptions around implementing remote blood pressure monitoring for hypertensive disorders of pregnancy: A survey-based study by Theepika Rajkumar, Sharon Hu, Annemarie Hennessy and Angela Makris in DIGITAL HEALTH
