# Supplementary material for: Clinician and patient perceptions around implementing remote blood pressure monitoring for hypertensive disorders of pregnancy: A survey-based study
Source: Digit Health. 2025 Jul 22;11:20552076251317567. doi: 10.1177/20552076251317567 (PMC12290385; doi:10.1177/20552076251317567)
Supplement: sj-docx-1-dhj-10.1177_20552076251317567 - Supplemental material for Clinician and patient perceptions around implementing remote blood pressure monitoring for hypertensive disorders of pregnancy: A survey-based study [file sj-docx-1-dhj-10.1177_20552076251317567.docx]

*Page 1 of 3*

**Mothers' perceptions of remote blood pressure monitoring**

In light of COVID-19 and natural disasters, we have turned to mobile technologies to address disruptions to healthcare access. Pregnancies at high risk of blood pressure disorders benefit from frequent monitoring of blood pressure. Remote monitoring of blood pressure is being explored to help this, but has not yet been integrated into standard practice.

Our study is interested in understanding mothers' perceptions towards remote monitoring of blood pressure. Thank you for taking part. You will firstly be asked for some personal details before being shown a series of statements for which you choose how much you agree, as well as one multi-choice and one short response question.

Any and all information you provide for this survey will remain completely confidential and will be de-identified during analysis. It will be stored within a secure university network. If you have any questions, please do not hesitate to contact.

Please complete the entire survey below.

Thank you!

How old are you?

__________________________________ (Please use the age you will be this year.)

What is your weight (in kg)?

__________________________________

((kg))

What is your height (in metres)?

__________________________________

((m))

BMI (auto-calculated):

__________________________________

What best describes your current relationship status?

Married

Unmarried but in a relationship

Not in a relationship

What do you do in daily life?

I am in school, I am studying

I work in paid employment

I am an independent entrepreneur

I am a housewife

I am unemployed

If you are in paid employment, do you have time off

Yes

work to get to your appointments?

No

*Page 2 of 3*

How many hours of time off do you get?

__________________________________

Which level of annual gross income best describes

$50,000

yours?

What is the highest level of education you have

Primary

completed?

Secondary

Certificate

Diploma

Bachelor's degree

Graduate diploma or certificate

Post-graduate degree

What is your ethnicity?

Aboriginal/Torres Strait Islander

North-West European

Southern or Eastern European

North African or Middle Eastern

South-East Asian

North-East Asian

Southern or Central Asian

People of the Americas

South or Central American

Sub-Saharan African

Pacific Islander

(

Please select the option that you most closely

identify with.)

| What is your first language? | __________________________________ |
| --- | --- |
| How many pregnancies have you ever had, including the current one and any that were lost? | __________________________________ |
| How many children do you have at home? | __________________________________ |
| How many pregnancies did not make it to term? (auto-calculated) | __________________________________ |
| How many weeks pregnant are you today? | __________________________________ |

What was the method of conception for your current

Natural conception

pregnancy?

IVF

Artificial insemination

Donated sperm

Donated eggs or embryos

List any medications you take, not including vitamins

or supplements.

__________________________________________

(If you don't take any, write 'N/A'.)

*Page 3 of 3*

**Tell us what you think about using mobile apps and recording blood pressure.**

| Strongly disagree | Somewhat disagree | Neutral | Somewhat agree | Strongly agree |
| --- | --- | --- | --- | --- |

Mobile apps are easy to use.

Mobile apps are time consuming to use.

I am intimidated by learning how to use a new app.

I am confident in recording my own blood pressure.

Mobile apps are safe and secure with private information.

I feel safe that only my healthcare staff can access my health information in remote monitoring of my blood pressure.

Remote monitoring of my blood pressure will help healthcare staff make better decisions about my care.

I would be happy to add remote monitoring of blood pressure to my care.

As I am at high risk, monitoring my blood pressure from home would not be as safe as monitoring it in the clinic.

What would make you hesitate to try remote monitoring

Difficult to use.

of blood pressure?

Time-consuming.

Poor accuracy/reliability.

Not secure or private with my health information.

Will not add to my care.

Will take away from my care.

(

Select as many or few answers as you think are

relevant.)

Are there any other reasons you would hesitate?

__________________________________________

(If not, write 'N/A'.)
