# Supplementary material for: Clinician and patient perceptions around implementing remote blood pressure monitoring for hypertensive disorders of pregnancy: A survey-based study
Source: Digit Health. 2025 Jul 22;11:20552076251317567. doi: 10.1177/20552076251317567 (PMC12290385; doi:10.1177/20552076251317567)
Supplement: sj-docx-2-dhj-10.1177_20552076251317567 - Supplemental material for Clinician and patient perceptions around implementing remote blood pressure monitoring for hypertensive disorders of pregnancy: A survey-based study [file sj-docx-2-dhj-10.1177_20552076251317567.docx]

*Page 1 of 3*

**Clinicians' and midwives' perceptions of remote blood pressure monitoring**

In light of COVID-19 and natural disasters, mobile technologies have shifted to the forefront for addressing disruptions to healthcare access. Pregnancies at high risk of hypertensive disorders benefit from early detection through the frequent monitoring of blood pressure. Remote monitoring methods have shown feasiblity, but are yet to be integrated into standardised obstetric practice.

Our study is interested in understanding clinicians' and midwives' perceptions towards remote monitoring of blood pressure. Thank you for taking part. You will firstly be asked for some personal details before being shown a series of statements for which you choose how much you agree, as well as one multi-choice and one short response question.

What is your role in the care of pregnant women?

Obstetrician

Obstetrician/Medical Physician

Midwife

Any and all information you provide for this survey will remain completely confidential and will be de-identified during analysis. It will be stored within a secure university network.

If you have any questions, please do not hesitate to contact.

Please complete the entire survey below.

Thank you!

How old are you? (years)

__________________________________

)

Please use the age that you will be this year.

(

What is your gender?

Female

Male

Non-binary

Prefer not to say

What state do you work in?

NSW

VIC

QLD

SA

WA

NT

TAS

New Zealand

Where is your practice based?

Metropolitan

Rural

Remote

How many years of experience have you had in the care

of pregnant women?

__________________________________

*Page 2 of 3*

If you are a doctor, what is your level of training?

Registrar

Consultant

What is your ethnicity?

Aboriginal/Torres Strait Islander

North-West European

Southern or Eastern European

North African or Middle Eastern

South-East Asian

North-East Asian

Southern or Central Asian

People of the Americas

South or Central American

Sub-Saharan African

Pacific Islander

(

Please select the option that you most closely

identify with.)

What is your first language?

__________________________________

**Tell us what you think about using mobile technologies and recording blood pressure.**

| Strongly disagree | Somewhat disagree | Neutral | Somewhat agree | Strongly agree |
| --- | --- | --- | --- | --- |

Mobile apps are easy to use.

Mobile apps are time consuming to use.

I am intimidated by learning how to use a new app.

Patients are able to record their own blood pressure accurately.

Mobile apps are safe and secure with private information.

I feel safe that only relevant healthcare staff will have access to my patients' health information in remote monitoring of their blood pressure.

Privacy is something that concerns me about remote technologies in healthcare.

Remote monitoring of blood pressure will help me make better decisions about my patients' care.

Remote monitoring of blood pressure will decrease my workload.

*Page 3 of 3*

Undertaking remote monitoring of blood pressure in high risk women is unsafe.

Which of these do you think could be barriers to Patients will find it difficult to use.

using remote monitoring of blood pressure? Healthcare staff will find it difficult to use.

Patients will find it time-consuming.

Healthcare staff will find it time-consuming.

Poor accuracy/reliability.

It's not private or secure with health information.

It won't add to care I provide.

It will take away from care I provide. (Select as many answers as you think are relevant.)

Are there any other barriers you can think of?

__________________________________________

(If not, write 'N/A'.)
